# Analysis of the predictive value of red cell distribution width (RDW) and hemoglobin-to-RDW ratio (HRR) on the prognosis of patients undergoing total laryngectomy

**DOI:** 10.3389/fonc.2025.1651738

**Published:** 2025-10-01

**Authors:** Zhixuan Fang, Kai Niu

**Affiliations:** Department of Otorhinolaryngology, the First Hospital of Jilin University, Chang Chun, China

**Keywords:** laryngeal cancer, red cell distribution width, hemoglobin-to-red blood cell distribution width, prognosis, survival

## Abstract

**Background:**

Systemic inflammatory markers, particularly pretreatment red cell distribution width (RDW) and hemoglobin to red cell distribution width ratio (HRR), have been associated with prognosis in several cancers. This study aimed to investigate the relationship between the preoperative RDW, HRR and clinicopathologic characteristics of patients undergoing total laryngectomy and their correlation with prognosis.

**Methods:**

The optimal cut-off values of RDW and HRR to the overall survival (OS) of patients were determined by the receiver operating characteristic (ROC) curves, which in turn divided the patients into high-value and low-value groups for further stratified analyses. Patient survival was analyzed using Kaplan-Meier survival curves. Additionally, univariate and multivariate Cox regression analyses were conducted to evaluate the predictive roles of RDW and HRR on the prognosis of patients following total laryngectomy.

**Results:**

The high RDW group demonstrated statistically significant associations with TNM clinical stage, cervical lymph node metastasis, and vascular infiltration (P < 0.05). Similarly, the low HRR group exhibited significant associations with gender, histologic grade, TNM clinical stage, cervical lymph node metastasis, and vascular infiltration (P < 0.05).The optimal cut-off values for predicting overall survival (OS) for patients, as determined by ROC curves, were 13.75 for RDW and 10.79 for HRR. Additionally, RDW emerged as an independent prognostic factor for OS in this population (HR = 3.060, 95% CI 2.222–4.215, P < 0.001).

**Conclusion:**

Preoperative RDW and HRR are prognostic risk factors for OS in patients undergoing total laryngectomy, with RDW serving as an independent predictor of prognosis.

## Highlights

- A single-center retrospective study found that the preoperative RDW parameter and the hemoglobin-to-RDW ratio (Hb-to-RDW ratio, HRR) were prognostic risk factors for survival in laryngeal cancer patients undergoing total laryngectomy.
- RDW and HRR are simple, easy to measure and inexpensive indicators that can provide a valuable diagnostic basis for the prognosis of laryngeal cancer patients.

## 1 Introduction

Laryngeal cancer presents a significant public health challenge in China, with rising incidence and mortality rates over recent decades.As one of the most common malignant tumors of the head and neck, it accounts for nearly one-third of all head and neck cancers (1) and has a significant impact on global health. In 2022, an estimated 103,216 individuals are projected to die from laryngeal cancer worldwide, with age-standardized incidence and mortality rates of 3.9 and 2.1 per 100,000, respectively (2). Total laryngectomy is the primary treatment for patients with locally advanced laryngeal cancer; however, it inevitably impacts laryngeal functions, including phonation, swallowing, and respiration. To enhance the quality of life for patients, it is crucial to investigate prognostic factors for individuals with middle and late stage laryngeal cancer and to implement appropriate preventive and therapeutic measures.

Biomarkers are defined as physiological characteristics that can be objectively assessed and serve as indicators of pathological processes, risk, and pharmacological responses to therapy (3, 4). Recent studies have shown that the red blood cell distribution width (RDW) parameter in routine blood counts is strongly associated with the prognosis of various tumors. Red blood cell distribution width (RDW) is a marker of changes in red blood cell size. RDW contributes to cancer development through inflammatory responses in the microenvironment and has diagnostic and prognostic value for various tumor types, including lung, liver, prostate, esophageal cancers, and chronic lymphocytic leukemia (5–10). It is also considered a biomarker potentially involved in endothelial dysfunction (11), oxidative stress, and inflammatory processes in vascular diseases (3, 12).Hemoglobin (Hb) is one of the most important indicators in routine blood tests, reflecting the human body’s ability to produce red blood cells while also assisting in the diagnosis and treatment of various diseases. Studies have shown that hemoglobin levels correlate with prognosis in the treatment of various tumors. Hb serves as a diagnostic indicator of anemia and is also utilized for prognostic assessment in patients with lung cancer (13). Low Hb levels are recognized as a prognostic marker in patients with oral squamous cell carcinoma (14). Additionally, combining Hb with RDW has been shown to correlate with prognosis in various tumors (6, 7). In this paper, we analyze the clinicopathological characteristics and prognostic data of patients who underwent total laryngectomy to explore the potential connection between RDW, the Hb/RDW ratio (HRR), clinicopathological characteristics, and survival, aiming to identify a more convenient, reliable, and cost-effective index for the diagnosis and treatment of laryngeal cancer.

## 2 Materials and methods

### 2.1 Patients and data

In this study, we collected cases of patients who underwent total laryngectomy at the Department of Otorhinolaryngology and Head and Neck Surgery at the First Hospital of Jilin University, from January 2015 to December 2021. The collected data included age, gender, smoking history, drinking history, history of hypertension and diabetes mellitus, tumor site, T-stage, clinical stage of TNM, degree of histological differentiation, vascular infiltration, Perineural infiltration, and metastasis to the cervical lymph nodes. Based on pathological and imaging findings, clinicopathological staging was performed according to the eighth edition of the TNM staging criteria for laryngeal cancer published by the American Joint Committee on Cancer (AJCC).Additionally, the red blood cell distribution width (RDW) and hemoglobin (Hb) levels were obtained from the patients’ routine peripheral blood test results within one week prior to surgery. The hemoglobin-to-RDW ratio (HRR) was calculated as the ratio of peripheral blood Hb to RDW. All included patients were newly treated individuals with complete clinicopathological data and comprehensive peripheral blood hematology parameters, and the final number of patients included in the study was 117.

Inclusion criteria: 1) Patients with primary laryngeal squamous cell carcinoma treated by total laryngectomy; 2) No distant metastasis detected in the preoperative examination; 3) Good general health without serious systemic diseases; 4) Complete results of clinicopathological data and preoperative hematology tests.

Exclusion Criteria: 1) Patients with previous malignant tumors in the head and neck region; 2) Patients who have undergone prior treatments such as radiotherapy or chemotherapy before the operation; 3) Patients with preoperative conditions related to blood transfusion, hepatic or renal insufficiency, or any blood or immune system disorders that may affect inflammatory index levels; 4) Patients who have taken anticoagulants, antiplatelet agents, hormones, or other medications that could influence blood test results within one month; 5) Patients with infectious diseases, immune system disorders, or hematological diseases; 6) Patients with incomplete follow-up data. The primary endpoint of this study was overall survival (OS), defined as the duration from the day of surgery to the date of death from any cause or the last follow-up visit. As this was a retrospective study, informed consent from patients was waived. This study was approved by the Ethics Committee of the First Hospital of Jilin University, under Ethics Approval No. (2025) and Pro-review No. (2025-076).

Methods Peripheral blood was collected from the fasting antecubital vein of all patients in the morning. Hematological analysis was conducted using the SYSMEX XN9000 with routine blood testing, calibration and quality control strictly performed according to the manufacturer’s instructions.

### 2.2 Statistical analysis

Statistical analyses were performed using IBM SPSS version 27.0 (IBM, Chicago, Illinois, USA). Count data were expressed as numbers and percentages. The chi-square test was employed for comparisons between groups. Kaplan-Meier survival curves were generated for survival analysis, and Log-rank tests were utilized to detect group differences. Cox proportional hazards models were employed for both univariate and multivariate regression analyses related to prognosis. A p-value < 0.05 was considered statistically significant. Survival curves and forest plots were created using R software (version 4.4.1). The area under the curve (AUC) was calculated using Receiver Operating Characteristic (ROC) analysis, where an AUC > 0.5 indicated predictive value. Confidence intervals for all p-values were two-sided, and a p-value < 0.05 was considered statistically significant.

## 3 Results

### 3.1 Baseline characteristics of study population

A total of 117 patients met the inclusion criteria for this study. We stratified the patients by age (median age 62 years), gender, smoking history, drinking history, hypertension, diabetes mellitus, tumor site, tumor T-stage, TNM clinicopathological stage, degree of histological differentiation, vascular infiltration, perineural infiltration, and metastasis to the cervical lymph nodes (see **Table 1**).

**Table 1:** Baseline characteristics of study population.

Variable	Count(Percentage %)
Gender	
Male	97 (82.9)
Female	20 (17.1)
Age group (years)	
≤62	55 (47.0)
>62	62 (53.0)
Smoking history	
No	42 (35.9)
Yes	75 (64.1)
Drinking history	
No	68 (58.1)
Yes	49 (41.9)
Hypertension	
No	94 (80.3)
Yes	23 (19.7)
Diabetes mellitus	
No	110 (94.0)
Yes	7 (6.0)
TNM clinical stage	
I-II	26 (22.2)
III-IV	91(77.8)
Tumor T-stage	
T1-T2	30(25.6)
T3-T4	87(74.4)
Tumor site	
Supraglottic	33 (28.2)
Glottic	79(67.5)
Subglottic	5 (4.3)
Histological grade	
Poorly differentiated	26 (22.2)
Moderately differentiated	83 (70.9)
Well differentiated	8 (6.9)
Cervical lymph node metastasis	
No	68 (58.1)
Yes	49 (41.9)
Vascular infiltration	
No	67 (57.3)
Yes	50 (42.7)
Perineural infiltration	
No	104 (88.9)
Yes	13 (11.1)

### 3.2 Determination of optimal cutoff values for RDW and HRR using ROC curve analysis

**Figure 1:**
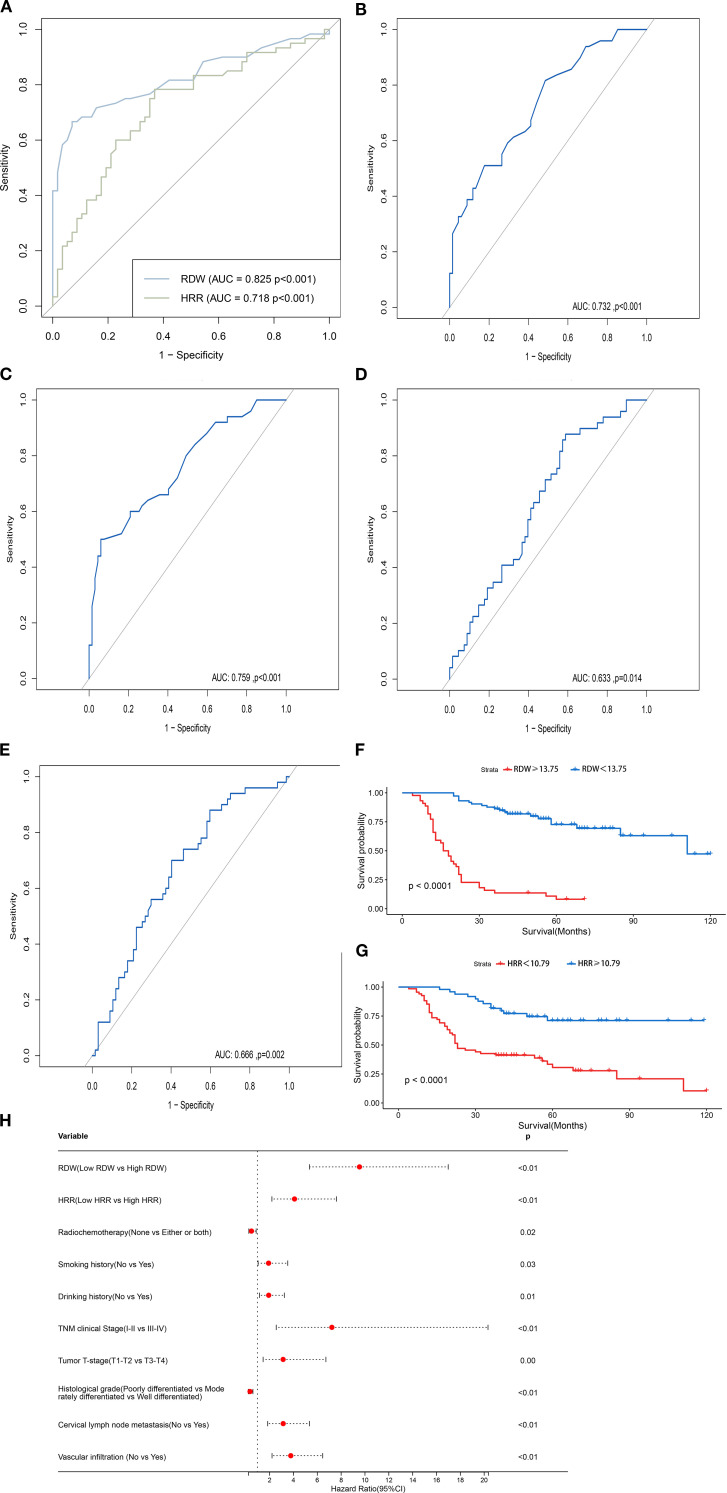
**(A)** The ROC curves of preoperative RDW and HRR cutoff values. **(B)** The ROC curve for RDW concerning cervical lymph node. metastasis. **(C)** The ROC curve for RDW concerning vascular infiltration. **(D)** The ROC curve for HRR concerning cervical lymph. node metastasis. **(E)** The ROC curve for HRR concerning vascular infiltration. **(F)** Survival curves for patients in high and low RDW. groups. **(G)** Survival curves for patients in high and low HRR groups. **(H)** HR Forest Plot for the Univariate Cox Proportional. Hazards Model of OS.

In this study, we assessed the predictive efficacy of RDW and HRR for the prognosis of patients undergoing total laryngectomy by comparing RDW and HRR values with their survival status, using the area under the curve (AUC) from ROC analysis. The optimal cutoff values were determined using the maximum Youden index (sensitivity + specificity - 1), and the ROC curves for RDW and HRR were generated (see **Figure 1A**). The AUC for RDW was 0.825 (95% CI: 0.748-0.902, P < 0.001), with a maximum Youden index of 0.596. The sensitivity and specificity were 66.7% and 92.9%, respectively, corresponding to an RDW value of 13.75. The AUC for HRR was 0.718 (95% CI: 0.625-0.812, P < 0.001), with a maximum Youden index of 0.415. The sensitivity and specificity were 78.3% and 63.2%, respectively, corresponding to an HRR value of 10.79 (see **Table 2**). The optimal cutoff values for stratification analysis were 13.75 for RDW and 10.79 for HRR. Patients were categorized into a low RDW group (RDW < 13.75) and a high RDW group (RDW ≥ 13.75), as well as a low HRR group (HRR < 10.79) and a high HRR group (HRR ≥ 10.79). We evaluated the predictive efficacy of RDW and HRR for the prognosis of patients undergoing total laryngectomy. This was accomplished by comparing RDW and HRR values with survival status using the area under the curve (AUC) from ROC analysis. The optimal cutoff values were determined using the maximum Youden index (sensitivity + specificity - 1), and the ROC curves for both RDW and HRR were generated (**Figure 1A**). The AUC for RDW was 0.825 (95% CI: 0.748-0.902, P < 0.001), with a maximum Youden index of 0.596. The sensitivity and specificity were 66.7% and 92.9%, respectively, corresponding to a cutoff value of 13.75 for RDW. For HRR, the AUC was 0.718 (95% CI: 0.625-0.812, P < 0.001), with a maximum Youden index of 0.415. The sensitivity and specificity were 78.3% and 63.2%, respectively, corresponding to a cutoff value of 10.79 for HRR (**Table 2**). In this study, optimal cutoff values of RDW (13.75) and HRR (10.79) were established for stratification analysis. Patients were classified into four groups: low RDW (RDW < 13.75), high RDW (RDW ≥ 13.75), low HRR (HRR < 10.79), and high HRR (HRR ≥ 10.79) (**Table 3**).

**Table 2:** Preoperative ROC curve analysis of RDW and HRR.

Index	AUC (95%CI)	Cutoff value	Youden index	Sensitivity (%)	Specificity (%)	P-value
RDW	0.825(0.748-0.902)	13.75	0.596	66.7%	92.9%	<0.001
HRR	0.718(0.625-0.218)	10.79	0.415	78.3%	63.2%	<0.001

**Table 3:** Relationship between RDW, HRR, and clinicopathological characteristics of laryngeal cancer.

Variable	RDW		X^2^	*p*	HRR		X^2^	*p*
	Low RDW	High RDW			Low HRR	High HRR		
	(N=73)	(N=44)			(N=68)	(N=49)		
Gender			0.562	0.453			4.745	0.029
Male	62(84.9%)	35(79.5%)			52(76.5%)	45(91.8%)		
Female	11(15.1%)	9(20.5%)			16(23.5%)	4(8.2%)		
Age (years)			1.608	0.205			0.131	0.717
≤62	31(42.5%)	24(54.5%)			31(45.6%)	24(49.0%)		
>62	42(57.5%)	20(45.5%)			37(54.4%)	25(51.0%)		
Smoking History			2.280	0.131			0.886	0.346
No	30(41.1%)	12(27.3%)			22(32.4%)	20(40.8%)		
Yes	43(58.9%)	32(72.7%)			46(67.6%)	29(59.2%)		
Drinking History			0.990	0.320			0.039	0.843
No	45(61.6%)	23(52.3%)			39(57.4%)	29(59.2%)		
Yes	28(38.4%)	21(47.7%)			29(42.6%)	20(40.8%)		
Hypertension			0.097	0.755			0.089	0.766
No	58(79.5%)	36(81.8%)			54(79.4%)	40(81.6%)		
Yes	15(20.5%)	8(18.2%)			14(20.6%)	9(18.4%)		
Diabetes Mellitus			0.259	0.611			0.713	0.399
No	68(93.2%)	42(95.5%)			65(95.6%)	45(91.8%)		
Yes	5(6.8%)	2(4.5%)			3(4.4%)	4(8.2%)		
TNM Clinical Stage			4.811	0.028			7.587	0.006
I-II	21(28.8%)	5(11.4%)			9(13.2%)	17(34.7%)		
III-IV	52(71.2%)	39(88.6%)			59(86.8%)	32(65.3%)		
Tumor T- Stage			2.058	0.151			2.174	0.140
T1-T2	22(30.1%)	8(18.2%)			14(20.6%)	16(32.7%)		
T3-T4	51(69.9%)	36(81.8%)			54(79.4%)	33(67.3%)		
Tumor Site			1.117	0.572			1.761	0.415
Supraglottic	21(28.8%)	12(27.3%)			17(25.0%)	16(32.7%)		
Glottic	50(68.5%)	29(65.9%)			49(72.1%)	30(61.2%)		
Subglottic	2(2.7%)	3(6.8%)			2(2.9%)	3(6.1%)		
Histological Grade			5.866	0.053			7.154	0.028
Poorly	11(15.1%)	15(34.1%)			19(27.9%)	7(14.3%)		
Moderately	57(78.1%)	26(59.1%)			42(61.8%)	41(83.7%)		
Well	5(6.8%)	3(6.8%)			7(10.3%)	1(2.0%)		
Cervical Lymph Node Metastasis			8.582	0.003			6.135	0.013
No	50(68.5%)	18(40.9%)			33(48.5%)	35(71.4%)		
Yes	23(31.5%)	26(59.1%)			35(51.5%)	14(28.6%)		
Vascular Infiltration			18.660	<0.001			9.046	0.003
No	53(72.6%)	14(31.8%)			31(45.6%)	36(73.5%)		
Yes	20(27.4%)	30(68.2%)			37(54.4%)	13(26.5%)		
Perineural Infiltration			0.005	0.946			0.742	0.389
No	65(89.0%)	39(88.6%)			59(86.8%)	45(91.8%)		
Yes	8(11.0%)	5(11.4%)			9(13.2%)	4(8.2%)		

### 3.3 Relationship between RDW, HRR, and clinical indicators of laryngeal cancer

Based on the optimal cutoff values for RDW and HRR, patients were categorized into two groups: low RDW (73 patients) and high RDW (44 patients), as well as low HRR (68 patients) and high HRR (49 patients).

The chi-square test indicated a significant correlation between the high RDW group and TNM clinical stage, cervical lymph node metastasis, and vascular infiltration. Additionally, there were statistically significant differences between the low HRR group and factors such as gender, histologic grade, TNM clinical stage, cervical lymph node metastasis, and vascular infiltration (P < 0.05) (**Table 4**).

**Table 4:** Univariate cox regression analysis of clinicopathological indicators.

Variable	Category	HR	95%CI	P
Gender	Male	ref.		
	Female	0.80	0.39-1.62	0.535
Age(years)	≤62	ref.		
	>62	0.85	0.51-1.42	0.554
Smoking History	No	ref.		
	Yes	1.94	1.06-3.55	0.031
Drinking History	No	ref.		
	Yes	1.95	1.17-3.26	0.010
Hypertension	No	ref.		
	Yes	0.61	0.30-1.25	0.173
Diabetes Mellitus	No	ref.		
	Yes	0.42	0.10-1.73	0.231
TNM Clinical Stage	I-II	ref.		
	III-IV	7.25	2.58-20.35	<0.001
Tumor T-Stage	T1-T2	ref.		
	T3-T4	3.16	1.48-6.75	0.003
Radiochemotherapy	None	ref.		
	Either or both	0.51	0.29-0.90	0.020
Tumor Site				0.250
	Supraglottic	ref.		
	Glottic	1.063	0.59-1.93	0.109
	Subglottic	2.474	0.82-7.49	0.840
Histological Grade				0.001
	Poorly differentiated	ref.		
	Moderately differentiated	4.94	0.68-35.99	0.115
	Well differentiated	11.80	1.58-88.33	0.016
Cervical Lymph Node Metastasis	No	ref.		
	Yes	3.2	1.87-5.37	<0.001
Vascular Infiltration	No	ref.		
	Yes	3.81	2.23-6.48	<0.001
Perineural Infiltration	No	ref.		
	Yes	1.22	0.55-2.69	0.626

Given the significant correlation between the high RDW and low HRR groups with cervical lymph node metastasis and vascular infiltration, the ROC curves for RDW and HRR concerning cervical lymph node metastasis and vascular infiltration were plotted, with cervical lymph node metastasis and vascular infiltration as the endpoints (**Figures 1B–E**). The AUC for the ROC curve calculated for cervical lymph node metastasis was 0.732 (95% CI, 0.641-0.823, P < 0.001), while the AUC for the ROC curve for vascular infiltration was 0.759 (95% CI, 0.671-0.848, P < 0.001), which indicate that RDW holds diagnostic value in predicting the presence or absence of cervical lymph node metastasis and vascular infiltration in patients undergoing total laryngectomy. The AUC for the ROC curve calculated for cervical lymph node metastasis was 0.633 (95% CI, 0.533-0.734, P = 0.014), while the AUC for the ROC curve calculated for vascular infiltration was 0.666 (95% CI, 0.567-0.764, P = 0.002),which indicate that HRR is equally valuable in predicting the presence of cervical lymph node metastasis and vascular infiltration in patients undergoing total laryngectomy.

### 3.4 Relationship between RDW, HRR, and prognosis of laryngeal cancer

As of the cut-off date for follow-up (December 31, 2024), patients who underwent total laryngectomy had a 1-year overall survival (OS) rate of 87.2% and a 3-year OS rate of 59.0%.

#### 3.4.1 RDW survival analysis

Patients in the low RDW group had a 1-year OS rate of 100.0%, significantly higher than the 3-year OS rate of 65.9% in the high RDW group. Additionally, the 3-year OS rate for the low RDW group was 84.9%, compared to 66.7% for the high RDW group. Survival curves for RDW were plotted using the Kaplan-Meier method (**Figure 1F**). The differences between the groups were compared using the Log-rank test. The results indicated that the OS in the low RDW group was significantly better than in the high RDW group (P < 0.001).

#### 3.4.2 HRR survival analysis

Patients in the high HRR group had a 1-year OS rate of 100.0%, significantly higher than the 77.9% rate in the low HRR group. The 3-year OS rate for the high HRR group was 81.6%, compared to 42.6% for the low HRR group. Survival curves for HRR were plotted using the Kaplan-Meier method (**Figure 1G**). The differences between the groups were compared using the Log-rank test. The results indicated that the OS in the high HRR group was significantly better than in the low HRR group (P < 0.001).

### 3.5 Univariate survival analysis of clinicopathological data

The univariate analysis incorporated clinicopathological indicators, revealing that RDW, HRR, smoking history, drinking history, tumor T-stage, TNM clinical stage, degree of histological differentiation, cervical lymph node metastasis, vascular infiltration, and postoperative radiochemotherapy status all significantly influenced OS in patients undergoing total laryngectomy (P < 0.05; **Figure 1H**).

### 3.6 Analysis of prognostic factors

The prognostic factors affecting survival in patients who underwent total laryngectomy included RDW, HRR, smoking history, drinking history, tumor T-stage, TNM clinical stage, degree of histological differentiation, cervical lymph node metastasis, vascular infiltration, and administration of postoperative radiochemotherapy.The positive results were incorporated into a multifactorial Cox proportional hazards model, which indicated that only RDW, histologic grade, smoking history, TNM clinical stage, and vascular infiltration were significantly associated with OS in patients who underwent total laryngectomy. RDW emerged as an independent prognostic factor for OS in patients undergoing total laryngectomy (HR = 3.060, 95% CI: 2.222-4.215, P < 0.001). Consequently, preoperative RDW was identified as an independent prognostic risk factor for patients undergoing total laryngectomy (P < 0.05) (**Table 5**).

**Table 5:** Multivariate survival analysis of clinicopathological indicators.

Variable	B	SE	Wald	*P*	HR	95%CI
RDW	1.118	0.163	46.883	<0.001	3.060	2.222-4.215
Histological Grade	-1.081	0.290	13.535	<0.001	0.339	0.191-0.603
TNM Clinical Stage	1.498	0.725	4.273	0.039	4.472	1.081-18.508
Smoking History	0.932	0.392	4.273	0.017	2.540	1.178-5.447
Vascular Infiltration	0.727	0.357	4.139	0.042	2.069	1.027-4.170

## 4 Discussion

The prevalence of laryngeal cancer is 1-2% among all cancer types (15). It is the second most prevalent malignant neoplasm of the upper respiratory system, following lung carcinoma (16). Every stage of tumor development is accompanied by an inflammatory response, in addition to raising the risk of cancer (17), chronic inflammation is closely linked to the onset of cancer. Inflammation and immune system activation are pivotal in tumor development, growth, and progression (18). RDW and HRR are measurable indices of systemic inflammatory response employed in the prognosis and diagnosis of diverse malignancies. Currently, no standardized criteria exist for their ideal cut-off levels. The principal techniques for ascertaining these values involve computations derived from ROC curves and crucial values defined in prior research.

In this study, the predicted values of RDW and HRR for OS in patients who underwent total laryngectomy for laryngeal cancer, as shown by our ROC curve, are 13.75 and 10.79, respectively. Medine Kara et al. discovered that elevated RDW increased postoperative mortality by 4.6 times in laryngeal cancer patients, with an acceptable cutoff value of 14.05 for death prediction based on ROC analysis (18). Other investigations revealed an appropriate cutoff value of 13.5% for tongue cancer (4). The found HRR value for nasopharyngeal cancer was 0.97 (19), which closely corresponds with the threshold value determined in our investigation. Factors like tumor kind, case quantity, treatment variability, and selection bias impede the standardization of a precise ideal cutoff value. Nonetheless, credible studies can provide a legitimate spectrum of alternatives for establishing acceptable RDW and HRR criteria.

In this study, we analyzed the clinical characteristics of two subgroups: the low RDW group (RDW < 13.75) and the high RDW group (RDW ≥ 13.75), as well as the low HRR group (HRR < 10.79) and the high HRR group (HRR ≥ 10.79). Our findings indicated that preoperative RDW was associated with TNM clinical stage, cervical lymph node metastasis, and vascular infiltration. Additionally, the preoperative HRR was significantly correlated with gender, histologic grade, TNM clinical stage, cervical lymph node metastasis, and vascular infiltration. Additionally, Medine Kara’s study indicated that RDW served as a predictor of postoperative mortality in laryngeal cancer, with elevated RDW increasing postoperative mortality by 4.6 times in these patients (18). Marcus K’s study revealed that elevated RDW was associated with nonspecific complications after laryngectomy, including deep vein thrombosis, pneumonia, cardiac events, and difficulties in extubation (20). Marcin Miszczyk’s research indicated that patients with RDW ≥ 13.5% had markedly reduced overall survival in comparison to those with RDW < 13.5%. The study determined that a pretreatment RDW of > 13.5% is a significant predictor of reduced overall survival in individuals with squamous cell carcinoma of the tongue (4).Yakup Bozkaya’s study demonstrated that low HRR was an independent variable affecting OS and disease-free survival in patients with locally advanced nasopharyngeal carcinoma (19).

These findings highlight the important role of RDW and HRR in evaluating the prognosis of patients with head and neck tumors, as well as their potential in predicting disease progression and overall patient outcomes. In this study, the RDW group showed a substantial correlation with TNM clinical stage, cervical lymph node metastasis, and vascular infiltration, with statistically significant differences noted. Statistically significant differences were identified between the HRR group and gender, histologic grade, TNM clinical stage, cervical lymph node metastasis, and vascular infiltration. The favorable outcomes from the univariate analysis were incorporated into the multivariate COX regression model. This investigation showed that RDW, histologic grade, smoking status, TNM clinical stage, and vascular infiltration were significantly associated with OS in patients undergoing total laryngectomy, with RDW identified as an independent prognostic factor for OS in this cohort. Consequently, this study concludes that RDW and HRR are straightforward, cost-effective, and readily identifiable markers. Additionally, this study is the inaugural publication that explicitly investigates HRR as a biomarker in the peripheral blood of patients undergoing total laryngectomy. This study indicates potential markers of laryngeal cancer progression, offering a valuable diagnostic basis for clinical practice; however, the prognostic predictive value of HRR is inferior to that of RDW.

Despite the association of increased RDW and low HRR with adverse prognostic outcomes, their precise mechanisms remain ambiguous. The correlation between erythrocytes, tumor prognosis, and immunological function is intricate and multifarious. In the tumor microenvironment, erythrocyte precursors can develop into myeloid cells that inhibit T-cell immunity (21). Concurrently, erythrocytes may potentially associate with cytokines that facilitate tumor proliferation and angiogenesis (22). Metabolic byproducts, such as lactic acid, might modify the pH of the tumor microenvironment, so indirectly affecting tumor growth (23). Moreover, direct interaction between erythrocytes and tumor cells might augment the invasive and migratory properties of malignancies (21). HRR, as a hematological indicator, denotes the ratio of HB to RDW. Low HRR values generally signify diminished hemoglobin levels and elevated RDW. This may be linked to chronic inflammation, which not only inhibits erythropoietin synthesis (24) but also modifies erythrocyte production and metabolism, hence reducing erythrocyte longevity (25, 26). Moreover, malnutrition, especially deficits in iron, folate, and vitamin B12, in conjunction with oxidative stress—which compromises erythrocytes and heightens their fragility—are substantial factors contributing to increased RDW. Hb, indicative of indicative of erythrocyte production capacity, frequently signifies a worse prognosis when levels are diminished. This process may arise from anemia leading to tumor hypoxia, which induces resistance to radiation and accelerates tumor angiogenesis.

However, this study has several limitations. Firstly, it is a retrospective case study that covers a restricted number of cases from a single center, hence impacting the generalizability of the findings. Secondly, HPV infection has been identified as a considerable risk factor for laryngeal cancer; however, HPV status was not assessed in this investigation due to absent data. In addition, the follow-up duration was somewhat short, and solely the OS metric was incorporated;disease-free survival, progression-free survival, and other critical prognostic markers in oncology patients were not considered. Consequently, additional prospective research with bigger sample numbers across other centers and geographies is essential to corroborate these findings in the future.

## 5 Conclusion

In conclusion, preoperative RDW and HRR are prognostic risk factors for overall survival (OS) in patients undergoing total laryngectomy, with RDW serving as an independent predictor of prognosis. Preoperative RDW showed significant associations with TNM clinical stage, cervical lymph node metastasis, and vascular infiltration, while preoperative HRR was significantly associated with gender, histologic grade, TNM clinical stage, cervical lymph node metastasis, and vascular infiltration. RDW and HRR, as simple and effective biomarkers, are closely associated with the occurrence and progression of laryngeal cancer, thereby providing clinicians with robust support in patient management and the development of treatment strategies.

## Data Availability

The raw data supporting the conclusions of this article will be made available by the authors, without undue reservation.
